# Exogenous chalcone synthase expression in developing poplar xylem incorporates naringenin into lignins

**DOI:** 10.1093/plphys/kiab499

**Published:** 2021-10-27

**Authors:** Elizabeth L Mahon, Lisanne de Vries, Soo-Kyeong Jang, Sandeep Middar, Hoon Kim, Faride Unda, John Ralph, Shawn D Mansfield

**Affiliations:** 1 Department of Wood Science, Faculty of Forestry, University of British Columbia, Vancouver, BC, Canada; 2 US Department of Energy, Great Lakes Bioenergy Research Center, Wisconsin Energy Institute, Madison, Wisconsin, USA; 3 Department of Biochemistry, University of Wisconsin, Madison, Wisconsin, USA

## Abstract

Lignin, a polyphenolic polymer, is a major chemical constituent of the cell walls of terrestrial plants. The biosynthesis of lignin is a highly plastic process, as highlighted by an increasing number of noncanonical monomers that have been successfully identified in an array of plants. Here, we engineered hybrid poplar (*Populus alba x grandidentata*) to express *chalcone synthase 3* (*MdCHS3*) derived from apple *(Malus domestica*) in lignifying xylem. Transgenic trees displayed an accumulation of the flavonoid naringenin in xylem methanolic extracts not inherently observed in wild-type trees. Nuclear magnetic resonance analysis revealed the presence of naringenin in the extract-free, cellulase-treated xylem lignin of MdCHS3-poplar, indicating the incorporation of this flavonoid-derived compound into poplar secondary cell wall lignins. The transgenic trees also displayed lower total cell wall lignin content and increased cell wall carbohydrate content and performed significantly better in limited saccharification assays than their wild-type counterparts.

## Teaser

Expressing exogenous, apple-derived chalcone synthase in actively lignifying poplar xylem tissue results in less total lignin, improved saccharification rates, and incorporation of naringenin into lignins.

## Introduction

Lignin, a major chemical constituent of lignocellulosic biomass, poses a significant barrier to the efficient industrial processing for the production of pulp and paper, specialty chemicals and fibers, and liquid biofuels. However, this complex polyphenolic polymer may serve as a chemical precursor in the development of new bio-based materials, high-value polymers, and chemicals. Although fast-growing woody feedstocks, such as poplar, willow, and eucalyptus, represent abundant and renewable sources of lignocellulosic biomass, narrow profit margins continue to limit the economic feasibility of employing them as dedicated energy crops at an industrial scale (Mahon and Mansfield, 2019).

Lignin is typically derived from three canonical monolignols: *p*-coumaryl, coniferyl, and sinapyl alcohols, which undergo oxidative coupling in the developing cell wall to form polymeric lignin. Efforts to genetically engineer the core monolignol biosynthetic pathway have led to significant changes in both content and composition of lignin, highlighting the remarkable metabolic plasticity of this biosynthetic pathway (Ralph et al., 2004; Leple et al., 2007; Coleman et al., 2008a, 2008b; Sykes et al., 2015; Chanoca et al., 2019). Moreover, a wide array of noncanonical monolignols has recently been found to naturally incorporate into lignins of different plant species (Vanholme et al., 2019). For example, the stilbenoid compounds, resveratrol, piceatannol, and isorhapontigenin, have all been identified as monomers in lignins of palm fruit endocarps (del Río et al., 2017), and their respective stilbene glycosides have also been identified in the lignins of Norway bark (Rencoret et al., 2019). Hydroxycinnamamides, specifically ferulamides, have been shown to incorporate into plant lignins, behaving as lignin monomers (Negral et al., 1996; del Río et al., 2020). Also, the high-value flavonoid tricin, reported to have a wide variety of potential pharmaceutical applications (Li et al., 2016), was found incorporated at the ends of lignins in many monocots (del Río et al., 2012; Lan et al., 2015). Recently, disruption of *flavone synthase II* (*fnsII*) in rice resulted in the accumulation of naringenin, a flavanone precursor to tricin, and the subsequent occurrence of naringenin in the lignin-enriched cell wall fraction, indicating that other flavonoids could be engineered into grass lignins as well (Lam et al., 2017).

**Figure 1:**
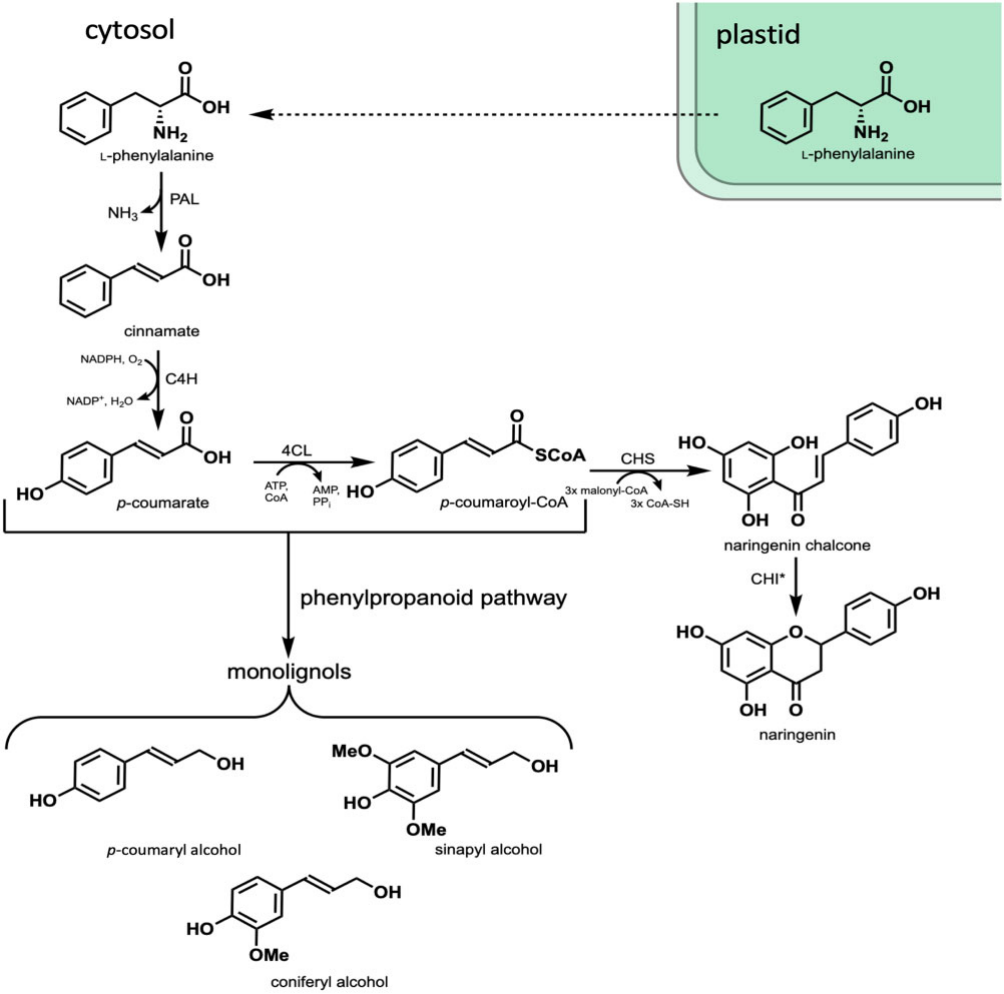
Biosynthesis of naringenin in xylem tissue. Phenylalanine is produced in the plastid via the shikimate pathway and transported into the cytosol where it is deaminated by phenylalanine ammonia-lyase to produce cinnamate, which is then hydroxylated by cinnamate 4-hydroxylase (C4H) producing *p-*coumarate. *p-*Coumarate is converted to *p-*coumaroyl-CoA by 4CL. CHS then combines three molecules of malonyl-CoA with *p-*coumaroyl-CoA producing naringenin chalcone, which is isomerized to (2*S*)-naringenin by CHI. *p-*Coumaroyl-CoA and *p-*coumarate are both important precursors in the biosynthesis of monolignols: *p-*coumaryl alcohol, coniferyl alcohol (CA), and sinapyl alcohol.

Chalcone synthase (CHS) catalyzes the first committed reaction in the production of flavonoid compounds by combining *p*-coumaroyl-coenzyme A (CoA), a precursor in the monolignol biosynthetic pathway, with three malonyl-CoA units to produce naringenin chalcone, which is then cyclized to naringenin (Figure  1). Previous genetic manipulations in plants have shown that the flavonoid and monolignol biosynthetic pathways are tightly linked. For example, RNAi-mediated silencing of an important monolignol biosynthetic gene, *hydroxycinnamoyl-CoA:shikimate hydroxycinnamoyl transferase*, in *Arabidopsis* has led to the accumulation of flavonoids (Hoffmann et al., 2004). Similarly, downregulation of a monolignol biosynthetic gene, *caffeoyl-CoA O-methyl transferase*, in alfalfa (*Medicago sativa* L.) resulted in the accumulation of isoflavonoids, the predominant class of flavonoids in legumes (Gill et al., 2018). Conversely, silencing of *CHS* in maize (*Zea mays*) resulted in drastically reduced levels of the flavonoids apigenin and tricin, yet caused a significant increase in total lignin content of leaves (Eloy et al., 2017). Taken together, these results indicate that CHS plays an important role in directing carbon flux between monolignol and flavonoid pathways. In poplar, transgenic downregulation of *4-coumarate:CoA* *ligase* (4CL), another key monolignol biosynthetic gene, resulted in a reduction of lignin and led to the accumulation of the naringenin and kaempferol in stem extractives of transgenic trees (Voelker et al., 2010). Notably, naringenin and kaempferol were not observed in extractives of wild-type (WT) stem tissue, indicating that *CHS* expression may be low in stem tissue (Voelker et al., 2010). To confirm this, we evaluated the expression of all six previously identified putative poplar *CHS* genes (Zavala and Opazo, 2015) in WT poplar trees used for genetic transformation (*Populus alba* *x grandidentata*). We determined that expression levels were considerably lower in xylem tissue compared to leaf tissue, where flavonoids are known to accumulate (Supplemental Figure S1; Tian et al., 2021). Thus, overexpression of *CHS* in lignifying tissues of poplar, which does not appear to contain high levels of flavonoids, may serve to reduce lignin by redirecting carbon flux away from monolignol biosynthesis while simultaneously producing high-value flavonoids that could be incorporated into the lignins of important potential bioenergy crops such as poplar, adding further value to woody feedstocks.

To this end, we have genetically engineered hybrid poplar (*Populus alba* x *grandidentata*) to express a previously characterized *CHS* gene (*MdCHS3*) derived from apple (*Malus* x *domestica*) using a xylem-specific promoter (Yahyaa et al., 2017). *MdCHS3* (accession number NM_001328985) was selected for expression in lignifying tissues as it shows high substrate affinity for *p-*coumaroyl-CoA relative to reported *K*m values for competing poplar enzymes in the lignin biosynthetic pathway (Wang et al., 2018). MdCHS3 was also reported to display greater substrate specificity for *p-*coumaroyl-CoA over cinnamoyl-CoA making it a good candidate for expression in poplar xylem (Yahyaa et al., 2017). Poplar expressing *MdCHS3* in xylem tissue (hereafter referred to as *MdCHS3*-poplar) clearly displayed an accumulation of naringenin in xylem methanolic extracts, not inherently observable in WT, and nuclear magnetic resonance (NMR) analysis revealed the incorporation of this flavonoid compound (a flavanone) into polymeric lignins. In addition, the highest-expressing *MdCHS3*-poplar lines displayed reduced total lignin, increased cell wall carbohydrate content, yet displayed no changes in growth or biomass compared to their WT counterparts and significantly improved saccharification efficiency after dilute acid pretreatment.

## Results

### Generation of transgenic poplar expressing *MdCHS3*

MdCHS3 was previously characterized and shown to display greater substrate specificity for *p-*coumaroyl-CoA compared to cinnamoyl-CoA, as well as high catalytic efficiency (Yahyaa et al., 2017). As such, *MdCHS3* was selected to drive the production of naringenin in the developing xylem of poplar. *MdCHS3* was isolated from golden delicious apple seedlings and inserted into a plant expression vector under the control of a lignin-specific promoter, *AtC4Hp*. The *AtC4Hp::MdCHS3* expression construct was then transformed into hybrid poplar using *Agrobacterium-*mediated transformation as previously outlined (Wilkerson et al., 2014). Successful transformants were confirmed by genomic screening, and expression levels in leaves were subsequently determined by reverse transcription-quantitative PCR (RT-qPCR). The five highest-expressing lines were then clonally propagated and transferred to the greenhouse for growth in soil (Supplemental Figure S2).

### *MdCHS3*-poplar accumulate naringenin in developing xylem tissue

**Figure 2:**
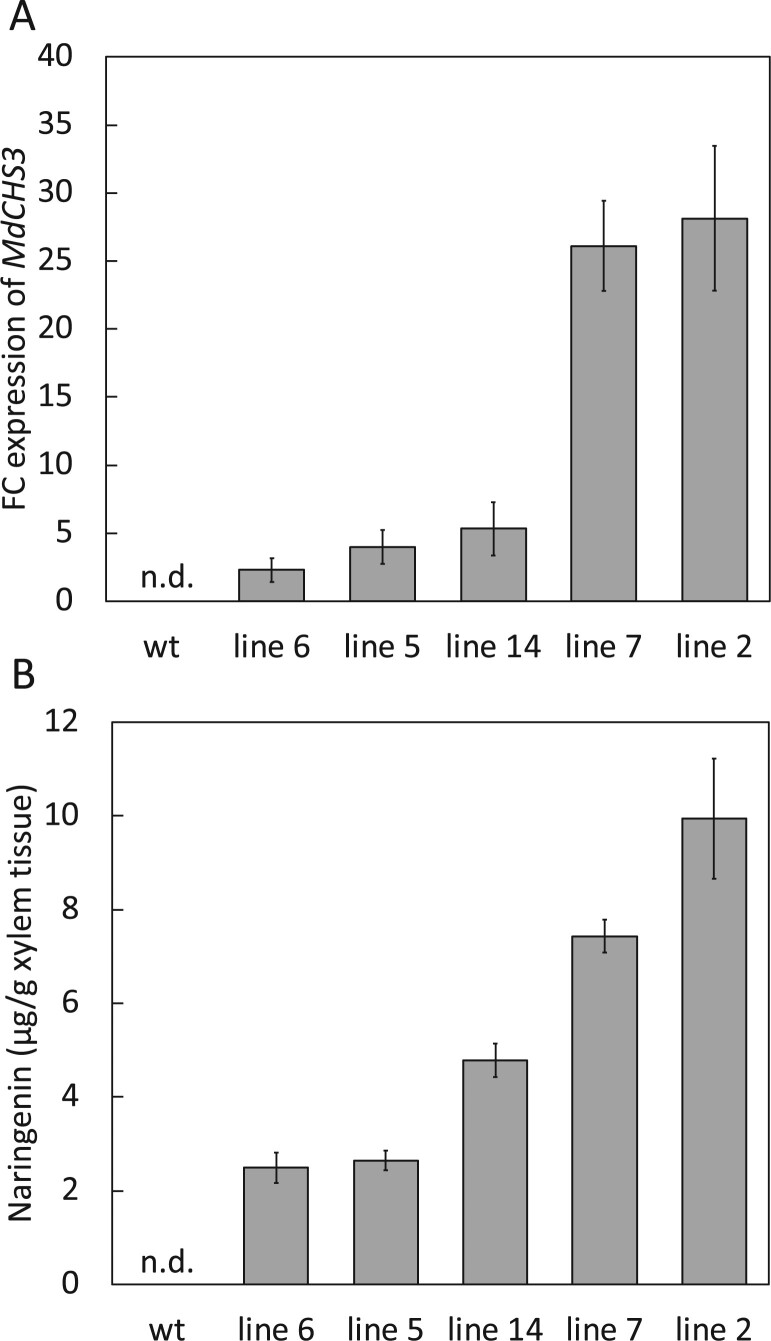
Expression of *MdCHS3* in poplar xylem tissue is associated with accumulation of naringenin in hydrolyzed methanolic extract. A, Relative expression levels of *MdCHS3* in transgenic poplar xylem tissue determined by RT-qPCR gene expression analysis are shown as fold change (FC) relative to the lowest expressing tree, originating from *MdCHS3*-poplar line 6. Expression of *MdCHS3* was not detectable in WT poplar. Expression levels represent the mean across five biological replicates, standard error indicated by error bars. B, Naringenin released after hydrolysis of methanolic extracts of *MdCHS3*-poplar xylem tissue. No naringenin was detectable in WT extracts (detectable levels >0.70 μg/g xylem tissue). Values represent the mean across five biological replicates for lines 5, 14, 7, and 2. Naringenin was detectable in only four of five biological replicates in line 6 (*n* = 4). Standard error indicated by error bars.

Trees were harvested after 16 weeks of growth, and expression of *MdCHS3* in mature xylem tissue was again confirmed via RT-qPCR analysis (Figure  2A). UPLC-DAD analysis of methanolic extracts from *MdCHS3*-poplar xylem tissue clearly revealed the accumulation of naringenin in its aglycone form, as well as multiple unknown compounds, accumulating in *MdCHS3*-poplar xylem and not observed in WT trees (Supplemental Figure S3). Subsequent hydrolysis of the methanolic extracts resulted in the release of additional naringenin aglycone, ostensibly freed from *O*-glycosylated forms (Supplemental Figure 3). We detected a total of 2.49 – 9.94 μg naringenin/g dried xylem tissue in the hydrolyzed methanolic extracts derived from *MdCHS3*-poplar lines, in which variability corresponds with the expression level of *MdCHS3* (Figure  2B). In comparison, no naringenin was detectable in the methanolic extracts of WT xylem after hydrolysis. *MdCHS3*-poplar displayed no significant differences in stem diameter or biomass compared to WT trees. Most transgenic lines also displayed no significant differences in height, with the exception of trees from line 14, which were significantly taller than their WT counterparts (Supplemental Table S1 and Supplemental Figure S4).

### *MdCHS3*-poplar exhibit changes to cell wall composition

Ectopic *MdCHS3* expression draws carbon away from the monolignol biosynthetic pathway by combining *p-*coumaroyl-CoA with three molecules of malonyl-CoA to produce naringenin chalcone (Figure  1). To further investigate the impact of *MdCHS3* expression on lignin biosynthesis, we performed Klason lignin analysis and thioacidolysis on dried, extract-free xylem tissue. The higher-expressing *MdCHS3-*poplar lines (lines 5, 14, 7, and 2) displayed significant reductions in acid-insoluble lignin, as low as 14.87% total cell wall content, compared to 16.72% in WT whereas no differences in acid-soluble lignin were observed (Table  1). The three highest-expressing *MdCHS3*-poplar lines (lines 2, 7, and 14) also contained significantly less total lignin, as low as 18.16% total cell wall content compared to 20.2% observed in WT trees (Table  1). Thioacidolysis indicated no significant differences in lignin syringyl:guaiacyl (S:G) ratio (Table  1).

**Table 1:** Cell wall composition of WT and *MdCHS3*-poplar xylem

	Lignin content (mg/100 mg)			Monomer composition	Percentage of Holocellulose	Percentage of Alpha cellulose
Poplar line	Acid-soluble lignin	Acid-insoluble lignin	Total lignin	S:G ratio		
WT	3.47 (0.23)	16.72 (0.12)	20.19 (0.30)	2.79 (0.05)	63.77 (0.62)	35.90 (0.36)
Line 6	3.56 (0.12)	16.04 (0.36)	19.60 (0.38)	2.71 (0.10)	**66.75 (1.00)**	37.20 (1.39)
Line 5	4.01 (0.15)	**16.15 (0.19)**	20.16 (0.20)	2.71 (0.09)	65.42 (0.69)	33.02 (1.53)
Line 14	3.35 (0.10)	**15.70 (0.17)**	**19.05 (0.22)**	2.74 (0.02)	**66.74 (0.98)**	37.53 (1.30)
Line 7	3.22 (0.21)	**14.94 (0.39)**	**18.16 (0.47)**	2.77 (0.03)	64.70 (0.76)	**36.24 (0.60)**
Line 2	3.42 (0.12)	**14.87 (0.27)**	**18.27 (0.30)**	2.65 (0.05)	64.50 (0.29)	**38.77 (1.06)**

Total lignin content measured in extract-free whole-cell wall material of WT control and *MdCHS3-*hybrid poplar xylem determined using Klason lignin analysis. Lignin monomeric composition was determined by thioacidolysis and relative % carbohydrate cell wall content was determined by holocellulose and alpha cellulose reactions. Values represent the mean across five biological replicates per line, standard error in brackets. Statistically significant differences compared to WT are bolded (*P* < 0.05) and were determined using Student’s t test.

Reductions in lignin are often associated with changes to cell wall carbohydrate content (Coleman et al., 2008a, 2008b; Van Acker et al., 2013; Sykes et al., 2015). High-performance liquid chromatography (HPLC) analyses of the individual cell wall carbohydrates released during secondary acid hydrolysis revealed a significant increase in glucose, as well as increases in galactose and rhamnose in the highest-expressing line (line 2) compared to WT (Supplemental Table S2). In order to ascertain if the increased glucose content was derived from cellulose or hemicellulose, alpha cellulose was determined. Significant increase in alpha cellulose was observed in two of the transgenic lines (Table  1). An examination of acetic acid released from the cell wall following saponification demonstrated a slight, but significant reduction in the cell wall acetate content of the highest-expressing *MdCHS3*-poplar line 2 compared to WT (Supplemental Table S3). Together these results indicate that expression of *MdCHS3* led to not only the production of naringenin in developing xylem tissue but also to significant alterations in cell wall composition.

Naringenin, along with other flavonoids, is reported to inhibit auxin transport, resulting in tissue-specific accumulation of auxin (Brown et al., 2001; Peer et al., 2004; Peer and Murphy, 2007). This accumulation of auxin in cambial tissue and developing xylem could potentially initiate tension wood formation, which in turn could manifest itself in altering the cell wall carbohydrate profile (Gerttula et al., 2015). To investigate this possibility, we examined stem sections from the highest-expressing *MdCHS3-*poplar line 2 for evidence of tension wood formation; however, transgenics exhibited no differences in vessel number, area, or width compared to WT trees, nor was there a notable increase in cellulose staining with calcofluor white in cross-sections, suggesting that the change to cell wall carbohydrates is not the result of tension wood formation (Supplemental Table S4 and Supplemental Figure S5).

### Naringenin is incorporated into *MdCHS3*-poplar lignins

**Figure 3:**
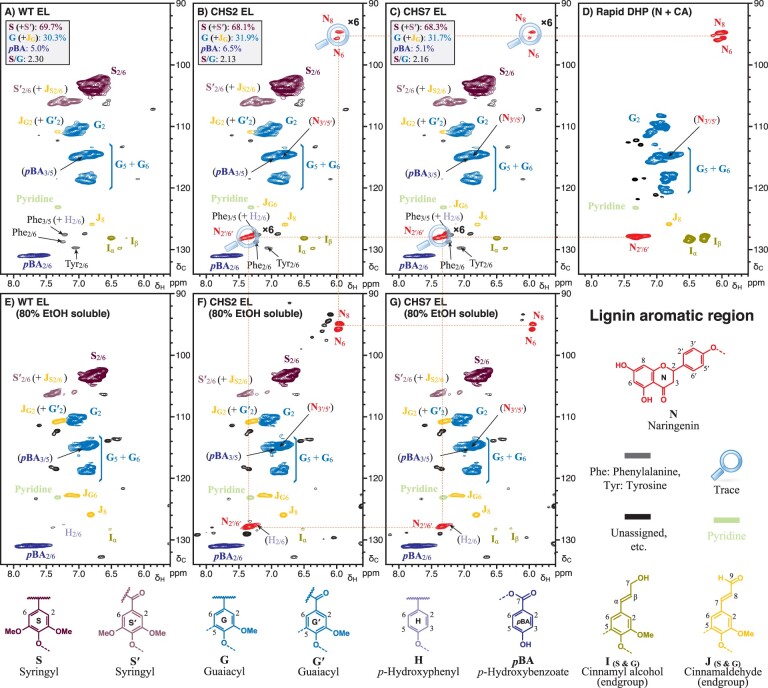
Analysis of aromatic region in 2D HSQC NMR spectra revealed the presence of naringenin in both the soluble extract and polymeric lignin fraction of *MdCHS3-popla*r xylem tissue. A–C, Cellulase-digested xylem EL fraction of WT and *MdCHS3*-poplar lines 2 and 7 xylem tissue. D, DHP prepared from naringenin and CA. E and F, Soluble fraction extracted with 80% ethanol from xylem tissue of WT and *MdCHS3*-poplar lines 2 and 7. Percentages represent the average across three biological replicates per line.

**Figure 4:**
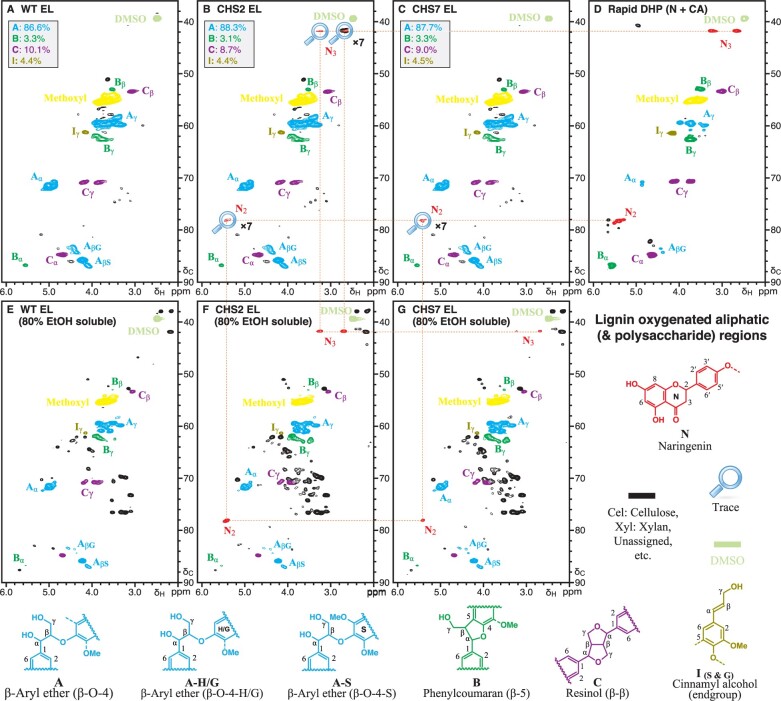
Analysis of aliphatic region in 2D HSQC NMR indicated the presence of naringenin in both the soluble extract and lignin fraction of *MdCHS3-*poplar xylem tissue. A–C, Cellulase-digested EL fraction of WT and *MdCHS3*-poplar lines 2 and 7 xylem tissue. D, DHP prepared from naringenin and CA. E and F, Soluble fraction extracted with 80% ethanol from xylem tissue of WT and *MdCHS3*-poplar lines 2 and 7. Percentages represent an average across three biological replicates per line.

Flavonoids, such as tricin, naturally incorporate into the lignins of monocot species such as grasses (del Río et al., 2012; Lan et al., 2016). Moreover, ^1^H–^13^C correlation (heteronuclear single-quantum coherence [HSQC]) NMR has identified naringenin in the lignin-enriched cell wall residue fraction of rice *fnsII* mutants with disrupted tricin biosynthesis (Lam et al., 2017). To determine whether naringenin is incorporated into the lignins of *MdCHS3-*poplar, we compared the lignin fraction (enzyme lignin [EL]) of WT and transgenic xylem tissue using 2D ^1^H–^13^C HSQC NMR. Analysis of the aromatic subregions revealed no substantial differences in canonical lignin components between WT and transgenic trees (Figure  3). However, trace signals at δ_C_/δ_H_ 94.8/5.98 (C_8_), 95.7/6.00 (C_6_), and 128.1/7.36 (C_2′/6′_) consistent with naringenin (or its phenolic ether) were observed in the lignin fraction of *MdCHS3-*poplar, but not in WT (Figure  3). The C_3′/5′_ peak at δ_C_/δ_H_ 115.0/6.88 of naringenin cannot be visualized in the lignin data because it is superimposed on one of normal G-unit peaks. We confirmed the presence of naringenin in transgenic lignins by comparing the spectra from the transgenic to that of a synthetic lignin polymer containing naringenin, which was prepared via an in vitro peroxidase-catalyzed polymerization (dehydrogenation polymer [DHP]) of naringenin (N) and coniferyl alcohol (CA) (Figure  3). In addition, trace signals at δ_C_/δ_H_ 78.3/5.45 and δ_C_/δ_H_ 41.8/3.27 and 2.70 corresponding to C_2_ and C_3_ of naringenin, respectively, were observed in the aliphatic subregions of both transgenic lignin fraction and synthetic N+CA lignin polymer HSQC spectra (Figure  4). No differences were observed in the polysaccharide anomeric subregions of WT and *MdCHS3*-poplar whole cell wall samples (Supplemental Figure S6). Finally, 2D HSQC NMR of 80% ethanol extracts clearly showed the presence of naringenin in *MdCHS3-*poplar xylem extracts but not in WT extracts (Figures  3 and 4).

### *MdCHS3*-poplars exhibit improved rates of limited saccharification

**Figure 5:**
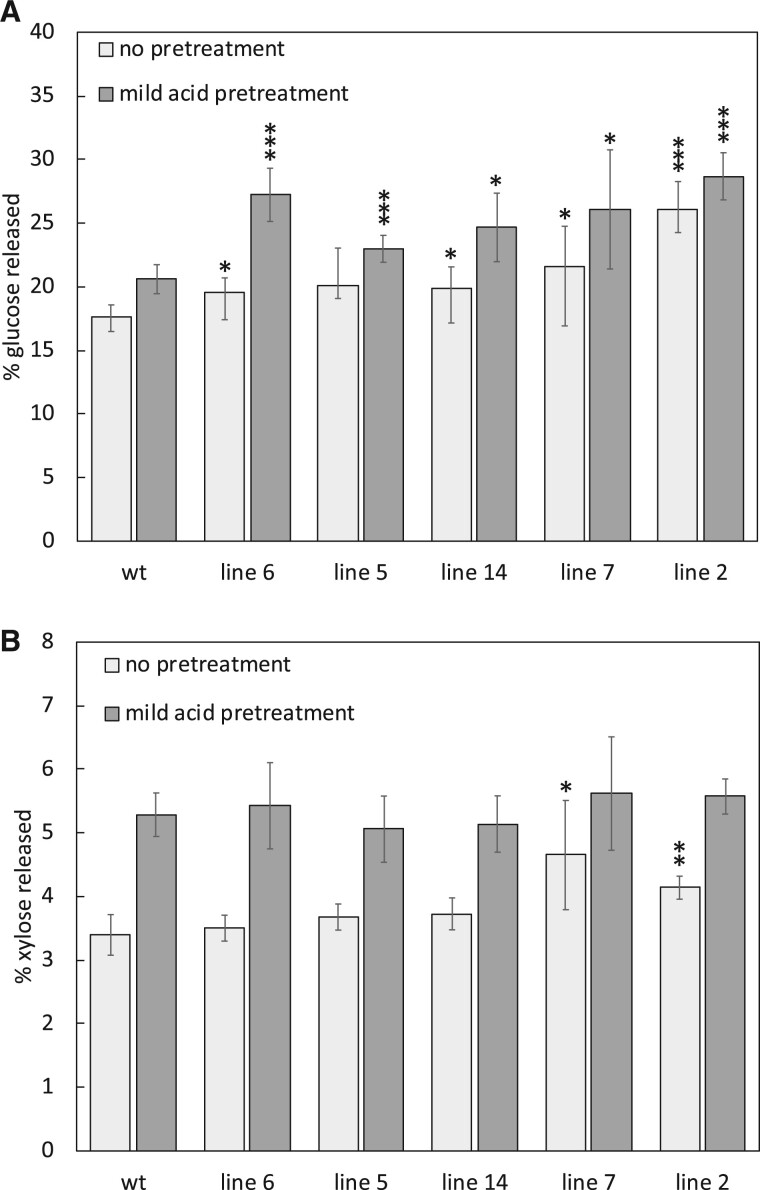
*MdCHS3*-poplar lines display significantly improved saccharification rates. A, Percentage of glucose released from nonpretreated and mild-acid-pretreated xylem tissue of WT and *MdCHS3*-poplar lines after 72 h saccharification. B, Percentage of xylose released during saccharification after no pretreatment and after mild-acid pretreatment. Values represent the mean taken across five biological replicates, two technical replicates each. Error bars represent standard deviation between biological replicates. Significant differences (^*^P < 0.1, ^**^P < 0.05, ^***^P < 0.01) compared to WT are starred and were determined using Student’s *t* test.

Reductions in total lignin have often resulted in improved rates of glucose and xylose release during bioconversion of lignocellulosic biomass, due to relative increases in cell wall polysaccharides and improved accessibility to both the cellulose and hemicellulose cell wall constituents by hydrolytic enzymes (Mooney et al., 1998; Berlin et al., 2005; Mansfield et al., 2012a, 2012b; Chanoca et al., 2019). To better understand the impact of reduced total lignin observed in *MdCHS3-*poplar combined with increased cell wall polysaccharides and incorporation of naringenin into lignins, we conducted a limited saccharification experiment on poplar wood both untreated and pretreated with dilute acid. Following 72-h of saccharification, glucose and xylose release reached a plateau (Supplemental Figure 7). All pretreated *MdCHS3* lines and four of the untreated *MdCHS3* lines released significantly more glucose after 72 h of saccharification (Figure  5A). Compared to the respective WT levels, line 2 released 48% more glucose when not pretreated and 39% more glucose when pretreated with a mild acid (Figure  5A). We also observed a significant increase in xylose released from the two highest-expressing lines compared to WT following no pretreatment, with line 7 releasing 21% more xylose than WT (Figure  5B). No increase in the released xylose was observed for any of the lines pretreated with a mild acid (Figure  5B), which is likely a function of the acid pretreatment employed prior to enzymatic hydrolysis that alone could facilitate the hydrolysis of the available xylan into its monomeric constituents.

## Discussion

Lignin is an important component of plant secondary cell walls, serving to facilitate water transport throughout the plant, support vertical growth, and protect against pests and pathogens (Weng and Chapple, 2010). Disruption of monolignol biosynthesis has led to significant reductions in lignin content and greatly improved biomass processability, yet these modifications often result in growth penalties (Coleman et al., 2008a, 2008b; Chanoca et al., 2019). This has motivated interest in genetic modifications of woody feedstock that specifically alter the composition of lignin, such as the incorporation of valuable monomers, as a method of improving lignocellulosic biomass (Mottiar et al., 2016; Mahon and Mansfield, 2019).

Expression of *MdCHS3* in hybrid poplar xylem resulted in an appreciable accumulation of naringenin in soluble extracts, in the form of glycosides, as well as accumulation of naringenin in the cell wall ostensibly incorporated into lignin. Poplar has been reported to produce naringenin endogenously in apical tissues, consisting of leaves, and three youngest internodia (Morreel et al., 2006; Morreel et al., 2006) as well as bud exudates (Greenaway et al., 1991). Low amounts of naringenin have even been reported in wood of mature poplar species both in its aglycone and glycosylated form (Pietarinen et al., 2006). However, in this study, naringenin was not observed in WT xylem extracts or EL of WT trees, nor does it appear to accumulate in stem tissue of younger trees (Voelker et al., 2010). In addition, we determined that expression levels of endogenous poplar *CHS* genes in WT xylem were substantially lower compared to *MdCHS3* in the lowest expressing transgenic line (line 6) which produced only trace amounts of naringenin in xylem (Supplemental Figure S1). Our data demonstrate that the expression of an exogenous *CHS* gene is sufficient to substantially increase production of naringenin in poplar xylem without introduction of an exogenous chalcone isomerase (*CHI*), the enzyme responsible for stereospecific ring closure of naringenin (Figure  1; Austin and Noel, 2003). The reduction in lignin observed in the highest-expressing lines could indicate that MdCHS3 is drawing significant carbon away from the biosynthesis of monolignols toward the production of naringenin chalcone. However, naringenin itself may play a role in suppression of lignin biosynthesis and contribute to the reduction in lignin observed in *MdCHS3*-poplar. Work in *Eucalyptus urograndis* demonstrated that root supplementation with naringenin altered lignin composition and resulted in downregulation of several lignin-related genes (Lepikson-Neto et al., 2013), and naringenin has been reported to directly inhibit the activity of 4CL, an important monolignol biosynthetic gene, in vitro (Voo et al., 1995).

Flavonoids have been found incorporated into the lignins of many grasses and other monocot species (del Río et al., 2012; Lan et al., 2016). 2D HSQC NMR analysis herein revealed the presence of naringenin in the lignin fraction of *MdCHS3-*poplar. To the best of our knowledge, this is the first report of a flavonoid compound being incorporated into poplar wood lignins as the result of genetic engineering. Naringenin was found to incorporate instead of tricin into the lignins of rice with disrupted *FNSII* expression via reactions occurring at the B ring resulting in 4′-O-β type coupling, which in turn results in β-aryl ether units, and 3′-β type coupling to produce phenylcoumaran units (Lam et al., 2017). Our data are consistent with previous NMR analyses of synthetic lignin polymers generated from radical coupling of naringenin with CA, which shows that the phloroglucinol ring remains intact, suggesting that coupling occurs mainly at the *p-*hydroxyphenyl B-ring over the phloroglucinol A-ring (Lam et al., 2017). This bears significance as the introduction of lignin monomers capable of single coupling reactions, such as naringenin, into lignifying tissue has been proposed as a strategy to reduce the length of lignin polymers and improve lignin solubilization during pretreatment processing (Eudes et al., 2012; Mottiar et al., 2016; Mahon and Mansfield, 2019). Due to the likely presence of 4′-O-β, 3′-β, and 3′-5 type linkages from the coupling of monolignols or lignin oligomers with naringenin, degradative methods such as thioacidolysis, which are targeted at cleaving β-ether linkages to release quantifiable monomers, would release only a fraction of the naringenin, that linked only by 4′-O-β linkages. Even for related tricin units that are incorporated into lignins via solely 4′-O-β linkages, the release was found to be 84% at best (Lan et al., 2016); from the original procedure (Rolando et al., 1992), thioacidolysis of poplar lignin releases typical lignin monomers accounting for just ∼24% of the total lignin. Considering that only trace amounts or naringenin were observed by NMR in the lignin fraction of MdCHS3-poplar xylem, thioacidolysis or any other current degradative technique would not be effective for quantifying the incorporation of naringenin into lignin polymers.

The occurrence of naringenin in the cell wall space raises questions concerning its export across the cell membrane. A comprehensive model describing the mechanisms of monolignol export from the site of synthesis to the cell wall space has yet to emerge (Perkins et al., 2019), although molecular simulations estimating membrane permeability of tricin indicated that passive diffusion alone is sufficient to facilitate transmembrane efflux (Vermaas et al., 2019). It is, therefore, possible that naringenin is similarly capable of passive diffusion across the membrane in its aglycone form; however, active transport by an unknown endogenous poplar transporter cannot be excluded. We also observed high levels of putative *O‒*linked naringenin glycosides in methanolic extracts and, considering that flavonoids are often stored in the vacuole in their glycosylated forms, naringenin may also be entering the cell wall space after release from the vacuole during programmed cell death (Zhao and Dixon, 2010; Perkins et al., 2019).

Some of the *MdCHS3*-poplar lines exhibited a significant increase in alpha cellulose cell wall content (Table  1). Analysis of cell wall carbohydrates released after hydrolysis indicated significant increases in glucose, galactose, and rhamnose in *MdCHS3* line 2 compared to WT trees (Supplemental Table S3). The significant improvement in glucose released during saccharification of *MdCHS3*-poplar lines is likely due to the combined relative increases in cell wall carbohydrates, including glucose, and the reduction in total lignin (Table  1; Supplemental Table S2). Lignin is thought to contribute to recalcitrance of lignocellulosic biomass by competitively binding cellulolytic enzymes and limiting access to cellulose (Mooney et al., 1998; Mansfield et al., 1999; Berlin et al., 2005), such that genetically modified trees with reduced lignin content often display drastically improved rates of saccharification (Leple et al., 2007; Mansfield et al., 2012a, 2012b; Sykes et al., 2015). Saccharification rates may also be influenced by the reduction in lignin polymer length, as naringenin is only capable of single coupling and therefore prevents any further polymerization once incorporated (Lam et al., 2017). Reductions in polymer length have been previously associated with improvements in saccharification, ostensibly by reducing cross-linking between lignins and cell wall polysaccharides, thereby improving accessibility of hydrolytic enzymes to both the cellulose and hemicellulose moieties (Eudes et al., 2012). In addition, reductions in cell wall acetyl content, as observed in *MdCHS3-*poplar lines, have also been shown to improve saccharification in hybrid aspen as acetylation is thought to restrict the accessibility of glycanases to cell wall polysaccharides (Pawar et al., 2017).

## Conclusion

By expressing *MdCHS3* in lignifying xylem tissue of hybrid poplar, we have produced transgenic trees with reduced total lignin content and increased cell wall carbohydrate content. These trees display substantially improved saccharification rates after both no pretreatment and dilute acid pretreatment. In addition, *MdCHS3-*poplar exhibits no differences in growth or biomass yield compared to WT and produces naringenin, a valuable flavonoid compound in xylem tissue. Moreover, we have identified incorporation of naringenin into the poplar wood lignins, demonstrating that if lignin-compatible flavonoid compounds can be produced in lignifying tissue of poplar, these compounds could be incorporated into lignin thereby potentially making them available on a high scale. Moving forward, *MdCHS3*-poplars represent a useful genetic background into which many additional flavonoid biosynthetic enzymes may be introduced in order to produce other valuable lignin-compatible flavonoid compounds.

## Materials and methods

### Construct development

The *35S* promoter sequence present in the pH7WGY2 Gateway (Invitrogen, Carlsbad, CA, USA) plant expression vector was replaced with a 2900-bp section of the *Arabidopsis thaliana C4H* promoter sequence (*AtC4Hp*) using digestion ligation cloning method. Briefly, *AtC4Hp* was amplified from pTkan-*pC4H::schl::qsuB* plasmid (Eudes et al., 2015) by PCR using primers: AtC4HP + SacI FWD: 5′-GTGAGCTCTCCCATATGGTCGACGGAATGAGAGACGAGAGC-3′ and AtC4HP + XbaI REV: 5′-GCTCTAGAGCGGCCGCCTGCAGGTCGACCTAGGGGGCGAGAGTAATTG-3′, producing a PCR amplicon with SacI and XbaI restriction digestions sites at 5′ and 3′ positions, respectively. The ph7WGY2 Gateway vector and *AtC4Hp* PCR amplicon were then digested with SacI/SpeI and SacI/XBal respectively, using the FastDigest protocol (Thermo Fisher Scientific, Waltham, MA, USA). Fragments were separated using gel electrophoresis, purified using the PureLink gel extraction kit (Invitrogen, Carlsbad, CA, USA), and ligated together with T4 DNA ligase (Thermo Fisher Scientific, Waltham, MA, USA) to generate the *AtC4Hp*-ph7WGY2 vector. *MdCHS3* (NM_001328985) was isolated from golden delicious apple seedlings (*Malus domestica*). Seeds were collected from locally purchased apples, stratified at 4°C in the dark for 2 weeks, and germinated on soil under long-day conditions. RNA was extracted from ground apple seedlings using the *Purelink* *plant RNA extraction kit* (Invitrogen, Carlsbad, CA, USA). Contaminating DNA was removed from RNA samples using the *TURBO DNA Free kit* (Invitrogen, Carlsbad, CA, USA), and 1 µg of RNA was used to synthesize cDNA with the *iScript cDNA Synthesis Kit* (Bio-Rad Laboratories, Hercules, CA, USA). The coding region of *MdCHS3* was amplified using BestTaq polymerase (Applied Biological Materials, Richmond, BC, Canada) from 1 µL of cDNA with primers modified to included Gateway *attB1* and *attB2* cloning sites at the 5′ and 3′ positions respectively: AttB1-MdCHS3 FWD: 5′-GGGGACAAGTTTGTACAAAAAAGCAGGCTATGGTGACCGTCGAGGAAGTT-3′ and AttB2-MdCHS3 REV: 5′-GGGGACCACTTTGTACAAGAAAGCTGGGTTCAAGCAGCCACACTGTGAAGCAC-3′. *MdCHS3* was cloned into the Gateway entry vector pDONR221 (Invitrogen, Carlsbad, CA, USA) by BP recombination and transferred via LR recombination into the *AtC4Hp*-pH7WGY2 vector generating the *AtC4Hp*::*MdCHS3* plant expression construct.

### Poplar transformations with *AtC4Hp::MdCHS3*

Transformation was performed as outlined in Wilkerson *et al.* 2014. *Populus alba x grandidentata* (P39) leaf discs were harvested from 6-week-old plants and co-cultivated with an *Agrobacterium tumefaciens* (EHA105) containing the *AtC4Hp*::*MdCHS3* expression construct. Following co-cultivation with *Agrobacterium* (OD_600_ _nm_ = 0.12) in woody plant media (WPM) for 30 min at 28°C in a gyratory shaker (100 rpm), the discs were blotted dry on sterile filter paper, and placed abaxially on WPM (0.1 µM of NAA, BA, and TDZ) culture medium. Plates were incubated in the dark for 2 d and transferred on the third day to WPM media (0.1 µM of NAA, BA, and TDZ) containing 250 mg/L cefotaxime and 500 mg/L carbenicillin to eliminate the *Agrobacterium.* Plates were incubated in the dark for two subsequent days then transferred to WPM (0.1 µM of NAA, BA, and TDZ) selection media containing 250 mg/L cefotaxime, 500 mg/L carbenicillin, and 20 mg/L hygromycin to select for successful transformants. After 5 weeks, discs were transferred to new WPM selection media (0.01 µM BA), and after emergence, shoots (one shoot per leaf disc) were transferred to WPM selection media (0.01 µM NAA). Plants were confirmed transgenic by screening genomic DNA after 6 weeks of growth. Genomic DNA was extracted using a cetyltrimethylammonium bromide (CTAB) (Sigma-Aldrich Co., St Louis, MO, USA) DNA extraction method and quantified using a spectrophotometer ND1000 (NanoDrop Technologies LLC, Wilmington, DE, USA). DNA was stored at −20°C. *MdCHS3* was detected using PCR screening of genomic DNA with gene-specific primers: MdCHS3-cds-FWD: 5′-ATGGTGACCGTCGAGGAAGTT-3′ and MdCHS3-cds-REV: 5′-GCAGCCACACTGTGAAGCAC-3′. Transgenic plants were sub-cultured, multiplied (minimum 8 replicates per transformation event), and transferred to antibiotic-free WPM media (0.01 µM NAA). After 4 weeks of growth, the tops of clonally propagated lines were excised and transferred to new antibiotic-free WPM media and grown in tissue culture. After 6 weeks of growth, leaves were collected from tissue culture, and RT-qPCR was used to determine *MdCHS3* transcript abundance.

### Growth conditions and measurements

Six-week-old plants, grown from tops, were transferred from WPM media into two-gallon pots containing perennial soil mix (50% peat, 25% fine bark, and 25% pumice; pH 6.0) and grown under 16 h supplemental light, and watered daily on flood tables at the UBC greenhouse. After 4 months of growth, height was recorded by measuring the distance from root collar to tree apex; stem diameter was determined using digital calipers 10-cm above the root collar. Weight of fresh biomass of the whole tree cut 10-cm above root collar collected immediately after cutting.

### Gene expression analysis

RNA was extracted from tissue culture leaves using TRIzol RNA extraction (Thermo Fisher Scientific, Waltham, MA, USA) and from freshly scraped poplar xylem tissue, and mature expanded leaves using a modified CTAB RNA extraction method to account for high quantities of phenolics present in xylem tissue of *MdCHS3-*poplar (Chang et al., 1993). cDNA was produced from extracted RNA as described above. Relative expression levels of putative poplar *CHS* genes were quantified using BlasTaq 2X qPCR MasterMix (Applied Biological Materials, Richmond, Canada). Reactions consisted of 5  µL 2X master mix, 20 pmol of primers, 1 µL of cDNA, and nuclease-free water to a volume of 10 µL. Relative expression levels were determined using the following primers: *Potri.014G145100-*qPCR-FWD: TGA TAC TCA CTT GGA TTC AA; *Potri.014G145100-*qPCR-REV: TGG AAG TGT CAG GAT CAG; *Potri.003G176800 & Potri.003G176700-*qPCR-FWD: CGA TAC CCA TCT TGA TAG CC; *Potri.003G176800 & Potri.003G176700-*qPCR-REV: CTC CTA CCA CTG GGT CAG; *Potri.001G051600-*qPCR-FWD: TGA CAC TCA CCT TGA TAG CC; *Potri.001G051600-*qPCR-REV: CCC CCA GCA CAG GAT CCG; *Potri.001G051500-*qPCR-FWD: TGA CAC CCA CCT CGA TAG TC; *Potri.001G051500-*qPCR-REV: CCC CCA GCA CAG GAT CCG; *Potri.003G176900-*qPCR-FWD: CGA TAC TCA TCT TGA TAG CC; *Potri.003G176900-*qPCR-REV: CTC CTA TCA CTG GAT CAG.

RT-qPCR was performed using the following cycling parameters: 1 cycle of 5 min at 94°C, 39 cycles of 94°C for 10 s, and 58°C for 30 s, 1 cycle of 94°C for 10 s, followed by a melt curve cycle of 56°C–95°C increment of 0.5°C for 5 s to ensure amplification of only one product. Reactions were performed in triplicate. Fold change (FC) ratio of gene target to reference *PtEF1-*β (Potri.009G018600) was determined with correction for primer efficiency. Relative expression levels of *MdCHS3* were quantified using SsoFast Eva Green Supermix (Bio-Rad Laboratories, Hercules, CA, USA) and the following primers: *MdCHS3-*qPCR-FWD: TGT CAA GTG CGT GTG TCT TG; *MdCHS3-qPCR-REV*: TCC AGT CCT TCT CCA GTT GTT. RT-qPCR reactions consisted of 5 µL of SsoFast Eva Green^®^ Supermix (Bio-Rad Laboratories, Hercules, CA, USA) 20 pmol of primers, 0.3 µL of cDNA, and nuclease-free water to a total volume of 10 µL. RT-qPCR was performed on a CFX 96 System (Bio-Rad Laboratories, Hercules, CA, USA). The 88-bp fragment of the *MdCHS3* transcript was amplified MdCHS3-qPCR primers following the cycle parameters listed above. Reactions were performed in triplicate. Relative transgene transcript levels were determined by first normalizing *MdCHS3 C*_q_ values to the average expression of *PtrTIFF (Potri.006G185000)* and *PtrUBQ (Potri.001G418500)* reference genes across all samples, then subtracting each sample’s normalized *MdCHS3*Cq from the individual tree with the highest reported *MdCHS3 C*_q_ and converting to fold-change difference relative to the lowest-expressing tree.

### Phenolic metabolite profiling

A section of fresh stem 10 cm from the root collar of each tree was cut, lyophilized for 24 h, and ground using a Wiley Mill (Thermo Fisher Scientific, Waltham, MA, USA) to pass through a 40-mesh screen and used in all downstream analysis. Five microliter *o-*anisic acid (2–3 mg/mL) was added to 25 mg of ground tissue as an internal standard. Two extractions were performed per sample. Tissue was incubated with 700 µL of 50% MeOH (0.01% [v/v] trifluoroacetic acid (TFA)) at 70°C for 15 min and centrifuged for 5 min at 13,000 rpm. Supernatant was collected and tissue was extracted twice more with 80% MeOH (0.01% TFA) and 100% MeOH (0.01% TFA). Supernatants from all three washes were pooled together, and 500-µL aliquot was taken for hydrolysis and combined with 25 µL 0.2 M NaOH. Samples were evaporated down to 10 µL in volume in a SpeedVac concentrator (Thermo Fisher Scientific, Waltham, MA, USA), mixed with 300 µL of 1 M HCl, and incubated at 95°C for 3 h. Phase extraction of phenolic compounds was performed by mixing with 400 µL of ethyl acetate, and the upper organic phase was collected, evaporated to dryness, and resuspended in 100% MeOH (0.01% TFA). Samples were analyzed on the Agilent 1290 Infinity II UPLC with the 1290 Infinity II Diode Array Detector (DAD) fit with an EclipsePlus C18 column (Agilent, St Clara, CA, USA). Naringenin was eluted from the column at 0.3 ml/min, using a gradient transition from 95% water (0.1% TFA): 5% acetonitrile to 60% water (0.1% TFA): 20% MeOH: 20% acetonitrile over 2 min, followed by a gradient transition to 50% MeOH: 50% acetonitrile over 6 and 2 min wash of 5% water (0.1% TFA): 95% acetonitrile. Naringenin was quantified using a standard curve generated from a dilution series of an external standard, and calculations were normalized to an internal standard, *o*-anisic acid. Naringenin was not detectable at levels >0.70 µg/g xylem tissue.

### Cell wall compositional analysis

The ground, lyophilized xylem powder samples were Soxhlet extracted with acetone for 24 h. The extractive-free material was used for all further cell wall analysis. Total lignin content was determined using a modified Klason lignin analysis as previously described (Coleman et al., 2006). Dried extractive-free tissue (200 mg), two reactions per sample, was treated with 3 mL 72% H_2_SO_4_ for 2 h, diluted to 3% H_2_SO_4_ with 112 mL of distilled water, then autoclaved at 121°C for 60 min. The mixture was filtered through a dry, pre-weighed medium coarseness crucible; the retentate dried overnight at 105°C, and acid-insoluble lignin was determined by weighing the retentate. The filtered aliquot was collected, and absorbance at 205 nm using a UV spectrophotometer was measured to determine acid-soluble lignin content. Carbohydrate content was determined by HPLC analysis of filtered aliquot as previously described (Huntley et al., 2003). Glucose, xylose, mannose, galactose, arabinose, and rhamnose were analyzed using Dx-600 anion-exchange HPLC (Dionex, Sunnyvale, CA, USA) on a CarboPac PA1 column at 1 mL/min and post-column detection. Concentration of sugars was determined using standard curves generated from a dilution series of external standards. Calculations were normalized to an internal standard, fucose. Lignin (S:G) monomer ratio was determined using thioacidolysis (Robinson and Mansfield, 2009) and analyzed using gas chromatography on a Thermo Trace 1310 instrument (Thermo Fisher Scientific, Waltham, MA, USA), equipped with an autosampler, FID detector, and a TG-5MS (30 m × 0.32 mm × 0.25 µL) capillary column.

Holocellulose and alpha-cellulose fractions were obtained using the methods described in Porth et al. (2013). Briefly, 150 mg of dried Soxhlet-extracted wood powder was combined with 3.5 mL of (60 mL glacial acetic acid + 1.3 g/L NaOH) and 1.5 mL of 20% sodium chlorite solution (20 g NaClO_2_ in 80-mL distilled water) and shaken at 50°C for 16 h. Reactions were quenched by placing tubes in an ice bath, the supernatant was removed, and the procedure was repeated. Reacted wood meal was filtered through pre-weighed coarse sintered-glass crucibles and washed with 50 mL of 1% glacial acetic acid, followed by 10 mL of acetone under applied vacuum. Crucibles were dried overnight at 50°C and weighed to determine holocellulose content. Alpha cellulose was obtained by reacting 2.5 mL (17.5% NaOH) with 30 mg holocellulose for 30 min, followed by the addition of 2.5 mL distilled water left to react for 30 min. Reaction mixtures were filtered through a fresh set of pre-weighed coarse sintered-glass crucibles and washed with 3 × 50 mL distilled water. The crucibles were then soaked in 1.0 M acetic acid for 5 min and washed with distilled water. Crucibles were dried overnight at 50°C and weighted to determine alpha-cellulose content.

### Acetyl content analysis

Cell wall acetyl content was determined via saponification of extractive-free xylem tissue. Extract free tissue (30 mg) was combined with 100 µL of butyric acid (1:20 dilution), used as an internal standard, and 1 mL of 2 M NaOH. Two reactions were performed for each sample. Samples were incubated at 30°C shaking at 500 rpm for 24 h. Reactions were acidified by adding 100 µL of 72% (12M) H_2_SO_4_ and placed on ice for 5 min. Samples were centrifuged at 13,000 rpm for 2 min, and the supernatant was collected and filtered through a 0.45-µm filter in preparation for HPLC analysis. Acetic acid released from saponification was determined on a Summit HPLC analytical system (Dionex, Sunnyvale, CA, USA) fit with an Aminex Ion exclusion HPX-87H column (Bio-Rad Laboratories, Hercules, CA, USA). Samples were eluted with 5% H_2_SO_4_ at a flow rate of 0.6 mL/min.

### NMR analysis

Dried stems were ground, extracted, and balled milled as previously described (Mansfield et al., 2012a, 2012b). Ground tissue extracted sequentially using sonication in 80% ethanol (3 × 20 min), acetone (1 × 20 min), chloroform–methanol (1:1 v/v, 1 × 20 min). Extract-free biomass was ball milled for 10 min followed by 10 min of rest for 3 h/500 mg of sample using a PM100 ball mill (Retsch, Newtown, PA, USA) vibrating at 600 rpm in zirconium dioxide vessels (50 mL) containing ZrO_2_ ball bearings (20 × 6.5 mm diameter). Ball-milled samples were digested four times over 3 d at 50°C using the commercial enzyme cocktails Cellic CTec3 and HTec2 (Novozymes, Bagsværd, Denmark) 30 mg/g in sodium citrate buffer (pH 5.0). The residual EL preparations were washed three times with deionized water and lyophilized overnight. Ball-milled whole cell wall, EL, and 80% ethanol extracts were analyzed by 2D ^1^H–^13^C HSQC NMR spectroscopy (Kim and Ralph, 2010; Mansfield et al., 2012a, 2012b; Kim et al., 2017).

### Limited saccharification assay

Pretreatment and limited saccharification assays were performed as previously described (Van Acker et al., 2013), with several modifications. Samples of ground, lyophilized xylem powder (15 mg) were subjected to either no pretreatment or a mild acid pretreatment, with two technical replicates per treatment. Samples subjected to mild acid pretreatment were incubated in 2% sulfuric acid at 80°C for 2 h. After incubation, the samples were neutralized and washed four times with water. The aliquots for saccharification without pretreatment were similarly washed four times with water. Both sets of samples were dried for 4 d at 50°C. Briefly, 1 mL of 0.1 M acetic acid buffer solution (pH 4.8) was added to wash samples and incubated at 50°C, shaking at 300 rpm. The enzyme Cellic CTec3 (Novozymes, Bagsværd, Denmark) was diluted 100 times, and 100 µL was added to each sample. After 4, 24, 48, and 72 h, 20 µL of aliquots were taken from the saccharification sample and diluted 10, 20, 20, and 20 times, respectively. The concentration of glucose and xylose in the diluted timepoint samples were determined by Dx-600 anion-exchange HPLC (Dionex, Sunnyvale, CA, USA) as described above.

### Microscopy

Stem samples from 4-month-old *MdCHS3*-poplar and WT poplar were soaked overnight in dH_2_O. Samples were cut into 30-µm cross-sections with a Spencer AO860 hand sliding microtome (Spencer Lens Co., Buffalo, NY, USA). Sections were treated with 0.01% Calcofluor-white in 1× PBS for 3 min, then washed 3 × 5 min in 1× PBS for cellulose staining (Falconer and Seagull 1985). To calculate average vessel diameter and number. Three trees from the highest expressing line, *MdCHS3-poplar* *line 2*, and WT were analyzed; pictures were taken from five different zones of each section. Vessels were counted in three separate images per tree at 2.5× magnification; vessel area and width of 25 vessels per tree were measured at 12.5× magnification. Sections were mounted and visualized with UV at 20× magnification. Sections were mounted and visualized with a Leica DRM microscope (Leica Microsystems, Wetzlar, Germany). Photos were taken with a QICam camera (Q-imaging, Surrey, BC) and analyzed with OpenLab 4.0Z software (PerkinElmer Inc., Waltham, MA, USA).

### Statistical analysis

Cell wall analyses, expression analysis, phenolic extractions, and saccharification analyses were carried out in technical duplicates, across five biological replicates (individually grown trees) from each line. Datasets were assessed for normality and equality of variance and significant difference compared to WT was determined using two-tailed *t* test at 95% confidence (*P* < 0.05). A mixed-effect model was used to account for batch variation in Klason hydrolysis of cell wall carbohydrates. A significant linear relationship between naringenin produced in developing xylem and exogenous expression of *MdCHS3* was determined using Pearson’s correlation coefficient for significance (*P* < 0.05).

### Accession numbers

*MdCHS3* (NCBI accession number NM_001328985) Putative poplar *CHS* genes (Potri.014G145100.1, Potri.003G176700.1, Potri.003G176800.1, Potri.003G176900.1, Potri.001G051500.1, Potri.001G051600.1)

## Supplemental data 

The following materials are available in the online version of this article.

**Supplemental Figure S1.** Relative expression of six putative endogenous *CHS* genes in poplar.

**Supplemental Figure S2.** Relative expression of *MdCHS3* in 10 independently transformed poplar plants.

**Supplemental Figure S3.** Naringenin glycosides in xylem extracts of MdCHS3-poplar line.

**Supplemental Figure S4.** *MdCHS3-*poplar growth attributes after 16 weeks of growth.

**Supplemental Figure S5.** Autofluorescence and calcofluor white staining of wild-type and *MdCHS*3 poplar stem tissue.

**Supplemental Figure S6.** 2D HSQC NMR spectra of whole-cell walls displaying polysaccharide anomeric region.

**Supplemental Figure S7.** Glucose and xylose released from xylem tissue during enzymatic saccharification.

**Supplementary Table S1.** Mean growth measurements of wild-type and *MdCHS*3-poplar trees after four months of growth.

**Supplementary Table S2.** Structural cell wall carbohydrates in xylem of wild-type and *MdCHS*3-poplar.

**Supplementary Table S3.** Cell wall acetate content in xylem tissue of wild-type and *MdCHS*3-poplar.

**Supplementary Table S4.** Vessel count and area in cross-sections of sixteen-week-old *MdCHS*3-poplar (line 2) and wild-type trees.

## Supplementary Material

kiab499_Supplementary_Data

## Notes

E.L.M. conceived the project, produced transgenic poplar lines, conducted wood chemistry analysis, and wrote the article with contributions from all the authors. L.d.V. conducted saccharification assays; S.-K.J. conducted holocellulose and alpha-cellulose analyses; S.M. aided in the isolation of *MdCHS3* and performed gene expression analyses; H.K. performed NMR analysis; F.U. provided technical assistance and guidance to E.L.M.; J.R. supervised completion of the manuscript; S.D.M. conceived the project, supervised the experiments and completion of the manuscript.

The author responsible for the distribution of materials integral to the findings presented in this article in accordance with the policy described in the Instructions for Authors (https://academic.oup.com/plphys/pages/general-instructions) is Shawn D. Mansfield (shawn.mansfield@ubc.ca).
