# Supplementary material for: Exogenous chalcone synthase expression in developing poplar xylem incorporates naringenin into lignins
Source: Plant Physiol. 2021 Oct 27;188(2):984–96. doi: 10.1093/plphys/kiab499 (PMC8825309; doi:10.1093/plphys/kiab499)
Supplement: kiab499_Supplementary_Data [file kiab499_supplementary_data.docx]

**Supplemental Materials**

**Exogenous chalcone synthase expression in developing poplar xylem incorporates naringenin into lignins**

Elizabeth L. Mahon^1,2^, Lisanne de Vries^1,2^, Soo-Kyeong Jang^1^, Sandeep Middar^1^, Hoon Kim^2^, Faride Unda^1,2^, John Ralph^2,3^ and Shawn D. Mansfield^1,2^.

^1^Department of Wood Science, Faculty of Forestry, University of British Columbia, Vancouver, BC, Canada

^2^US Department of Energy, Great Lakes Bioenergy Research Center, Wisconsin Energy Institute, Madison, WI, USA

^3^Department of Biochemistry, University of Wisconsin, Madison, WI, USA.

**Supplemental Figure S1:** Relative expression of six putative endogenous poplar *CHS* genes in poplar. Leaves (A) and xylem (B) tissue of WT poplar. Expression of *MdCHS3* in the lowest (line 6) and highest (line 2) expressing transgenic poplar shown for comparison. Relative expression determined by qRT-PCR and shown as fold change (FC) relative to the reference gene (*PtEF1-*$\beta$*)*. Error bars represent standard error across four biological replicates.

**Supplemental Figure S2:** Relative expression of *MdCHS3* in ten independently transformed poplar plants. Tissue collected from leaves in tissue culture. The five highest expressing individuals (dark grey) were selected for clonal propagation and growth in greenhouse. Relative expression determined by qRT-PCR and shown as fold change (FC) relative to the lowest expressing plant (line 9), error bars represent variation across three technical replicates.

**Supplemental Figure S3:** Naringenin glycosides in xylem extracts of MdCHS3-poplar line. UPLC analysis of xylem methanolic extracts display clear differences between wild-type (blue) and the highest expressing *MdCHS3*-poplar line 2 (green). Xylem methanolic extracts (A), hydrolysed methanolic extracts (B). Naringenin standard (purple).

**Supplemental Figure S4:** *MdCHS3-*poplar growth attributes after sixteen weeks of growth. Photos of individuals representing each of the five *MdCHS3-*poplar lines next to a WT tree.

**Supplemental Figure S5:** Autofluorescence and calcofluor white staining of stem tissue. Autofluorescence (A-C) and calcofluor white (D-F) staining of wild-type (A,D), *MdCHS3*-poplar line 2 (B,E) and *MdCHS3*-poplar line 7 (C,F). Transgenic lines exhibit no differences in vessel size and number and no increase in cellulose staining with calcofluor white (Magnification 20x, Scale bars= 50 $\mu m$).

**Supplemental Figure S6:** 2D HSQC NMR spectra of whole cell walls displaying polysaccharide anomeric region. Common polysaccharide nomenclature is used for peak identification.

**Supplemental Figure S7:** Glucose and xylose released from xylem tissue during enzymatic saccharification. Percentage of glucose released from xylem tissue after no pretreatment (A). Percentage of xylose release after no pretreatment (B). Percentage of glucose released after mild acid pretreatment (C). Percentage of xylose released after mild acid pretreatment (D). Percentages displayed as the mean across four biological replicates, two technical replicates each.

**Supplementary Table 1.** Mean growth measurements of wild-type and *MdCHS3*-poplar trees after four months of growth. Values represent the mean across five biological replicates per line. Standard error is represented in brackets. Significant differences (p<0.05) compared to wild-type are bolded and were determined using Student’s t-test.

| Poplar line | Height (cm) | Stem diameter (cm) | Fresh biomass (g) |
| --- | --- | --- | --- |
| WT | 174.6 (4.87) | 3.7084 (0.18) | 472.8 (48.36) |
| line 6 | 174.2 (9.69) | 3.3528 (0.28) | 409.4 (71.27) |
| line 5 | 182.2 (5.01) | 3.302 (0.11) | 393.2 (38.085) |
| line 14 | **192.6 (2.66)** | 3.7084 (0.29) | 471.8 (28.52) |
| line 7 | 183.0 (3.55) | 3.556 (0.16) | 413.4 (20.76) |
| line 2 | 179.4 (8.70) | 3.2004 (0.21) | 375.8 (54.05) |

**Supplementary Table 2.** Structural cell wall carbohydrates in xylem of wild-type and *MdCHS3-*poplar. Values represent the mean (μg/mg extracted tissue) across five biological replicates per line. Standard error is represented in brackets. Means were estimated from a mixed-effect model controlling for differences across four separate hydrolysis batches of twenty-four reactions each. Significant differences compared to wild-type are bolded (p<0.05) and determined using Student’s t-test.

|  |  | | | | | |  |
| --- | --- | --- | --- | --- | --- | --- | --- |
|  | Structural carbohydrates (μg/mg) | | | | | | |
| Poplar line | Arabinose | Rhamnose | Galactose | Glucose | Xylose | Mannose | Total carbohydrates |
| WT | 3.35 (0.13) | 4.18 (0.14) | 7.53 (0.76) | 432.97 (8.49) | 174.45 (5.89) | 10.05 (1.12) | 638.22 (12.59) |
| line 6 | 3.36 (0.14) | 4.62 (0.14) | 8.98 (0.76) | 434.87 (7.76) | 170.62 (5.89) | 11.97 (1.16) | 643.53 (12.59) |
| line 5 | 3.58 (0.14) | 4.69 (0.14) | 8.58 (0.77) | 450.41 (7.93) | 175.69 (5.95) | 13.39 (1.13) | 656.05 (11.85) |
| line 14 | 3.51 (0.13) | 4.51 (0.14) | 8.56 (0.76) | 442.91 (7.76) | 172.95 (6.20) | 9.68 (1.12) | 646.85 (11.65) |
| line 7 | 3.95 (0.14) | 4.67 (0.15) | 9.00 (0.81) | 444.28 (8.48) | 182.55 (6.20) | 9.00 (1.16) | 653.27 (12.58) |
| line 2 | 3.54 (0.13) | **4.91 (0.14)** | **10.51 (0.76)** | **466.58 (7.75)** | 198.47 (5.89) | 11.21 (1.12) | 670.00 (11.64) |

**Supplementary Table 3.** Cell wall acetate content in xylem tissue of wild-type and *MdCHS3-*poplar. Values represent the mean % acetate released from extract free cell wall material by saponification across five biological replicates per line. Standard error is represented in brackets. Significant differences were detected between *MdCHS3*-poplar line 2 and wild-type poplar, as determined by Student’s t-test (p<0.05).

| Poplar line | % Acetate |
| --- | --- |
| WT | 5.88 (0.13) |
| line 6 | 5.90 (0.04) |
| line 5 | 5.69 (0.13) |
| line 14 | 5.77 (0.20) |
| line 7 | 5.64 (0.21) |
| line 2 | **5.59 (0.11)** |

**Supplementary Table 4.** Vessel count and area in cross-sections of sixteen-week-old *MdCHS3*-poplar (line 2) and wild-type trees. Values represent the mean across three biological replicates with standard error represented in brackets. A total of fifteen vessels per biological replicate were measured for area and width. No significant differences were detected between *MdCHS2*-poplar line 2 and wild-type, as determined by Student’s test (p<0.05).

| Line | Area per vessel ($\mu$m^2^) | Width ($\mu$m^2^) | Vessel count (per 100 000 $\mu$m^2^) |
| --- | --- | --- | --- |
| WT | 1531.37 (324.1(14(4) | 47.01 (6.18) | 15.91 (1.85) |
| line 2 | 1578.64 (335.17) | 50.01 (6.14) | 14.74 (1.23) |
